# Evidence of Dopaminergic Processing of Executive Inhibition

**DOI:** 10.1371/journal.pone.0028075

**Published:** 2011-12-05

**Authors:** Rajendra D. Badgaiyan, David Wack

**Affiliations:** 1 Department of Psychiatry, State University of New York at Buffalo, Buffalo, New York, United States of America; 2 Department of Radiology, Harvard Medical School, Boston, Massachusetts, United States of America; 3 Department of Psychiatry, Veterans Affairs Medical Center, Buffalo, New York, United States of America; 4 Department of Nuclear Medicine, State University of New York at Buffalo, Buffalo, New York, United States of America; French National Centre for Scientific Research, France

## Abstract

Inhibition of unwanted response is an important function of the executive system. Since the inhibitory system is impaired in patients with dysregulated dopamine system, we examined dopamine neurotransmission in the human brain during processing of a task of executive inhibition. The experiment used a recently developed dynamic molecular imaging technique to detect and map dopamine released during performance of a modified Eriksen's flanker task. In this study, young healthy volunteers received an intravenous injection of a dopamine receptor ligand (^11^C-raclopride) after they were positioned in the PET camera. After the injection, volunteers performed the flanker task under Congruent and Incongruent conditions in a single scan session. They were required to inhibit competing options to select an appropriate response in the Incongruent but not in the Congruent condition. The PET data were dynamically acquired during the experiment and analyzed using two variants of the simplified reference region model. The analysis included estimation of a number of receptor kinetic parameters before and after initiation of the Incongruent condition. We found increase in the rate of ligand displacement (from receptor sites) and decrease in the ligand binding potential in the Incongruent condition, suggesting dopamine release during task performance. These changes were observed in small areas of the putamen and caudate bilaterally but were most significant on the dorsal aspect of the body of left caudate. The results provide evidence of dopaminergic processing of executive inhibition and demonstrate that neurochemical changes associated with cognitive processing can be detected and mapped in a single scan session using dynamic molecular imaging.

## Introduction

Neurochemical control of executive inhibition remains uninvestigated because of the lack of a reliable technique to detect task-induced changes in the brain chemistry. Indirect evidence acquired in cognitive studies suggests that dopamine may be involved in the processing. These studies have found that the patients with dysregulated dopamine neurotransmission show impaired performance in executive inhibition tasks. Thus, poor performance is reported in patients with attention deficit hyperactivity disorder (ADHD), Tourette's syndrome (TS), Parkinson's disease (PD), and schizophrenia [1]. In most of these studies modified Eriksen's flanker task [2] was used to elicit executive inhibition. Involvement of dopamine in the processing is suggested also by the data obtained in laboratory animals. For example, it was shown in monkeys that the number of inhibited neurons reduces significantly after depletion of dopamine [3]. The depletion therefore increases the number of nonspecifically activated neurons and reduces signal to noise ratio. As a result, the depleted monkeys find it extremely difficult to inhibit competing options and select an appropriate response.

Additionally, neuroimaging experiments have consistently reported increased activation in the brain areas that are innervated by dopaminergic neurons. In an fMRI experiment [4] we observed increased BOLD activation in the caudate, anterior cingulate cortex (ACC), and superior and middle frontal gyri during performance of the flanker task. Since these structures are innervated by dopamine, the experiment provides indirect evidence of dopaminergic processing of the inhibition. A number of neurocognitive models of learning (based primarily on the data acquired in laboratory animals) also assume involvement of dopamine in the processing. For example, the actor-critic model of reinforcement learning [5] assumes that dopamine-mediated processes help animals learn the most rewarding action by inhibiting competing options.

There is however no direct evidence of dopaminergic processing of the human executive inhibition. Because of the lack of direct evidence, its significance remains unclear. As a result, we have incomplete understanding of the neurocognitive deficits that are responsible for impaired inhibitory control in psychiatric and neuropsychiatric conditions.

In this experiment we used a newly developed dynamic molecular imaging technique [6], [7] to detect and map dopamine released during performance of a task of executive inhibition. The technique exploits the competition between dopamine and its ligand for receptor occupancy and detects dopamine released during task performance in a single scan session. We used this technique previously to study dopamine released during performance of a number of cognitive, emotional and behavioral tasks [6], [7], [8], [9], [10], [11], [12]. In the present experiment we detected and mapped dopamine released in the Congruent and Incongruent conditions of a modified Eriksen's flanker task [2]. The task elicited executive inhibition.

## Results

In the modified Eriksen's flanker task performed in the PET camera, volunteers made accurate responses in most trials. In the Congruent condition they made 97.1±2.0% correct response while in the Incongruent condition 91.0±10.9% of responses were accurate. Even though responses were less accurate in the Incongruent condition, the accuracy was not significantly different from that in the Congruent. Similarly, response time was longer in the Incongruent (695±183 msec) as compared to the Congruent (602±151 msec) condition but the difference was not significant statistically. The trend of lower accuracy and greater response time in the Incongruent condition indicated cognitive cost of processing the inhibition. For making a response in this condition volunteers had to inhibit prepotent responses indicated by the direction of flanker arrowheads. This inhibition was not needed in the Congruent condition in which target and flanker arrowheads pointed to the same direction.

As described in Materials and Methods section, analysis of the PET data involved measurement of a number of receptor kinetic parameters using two models: linear extension of simplified reference region model or LE-SRRM [12], [13]; and extended simplified reference tissue model or E-SRTM [14]. Using the LE-SRRM we dynamically measured changes in the rate of ligand displacement (γ) in the Incongruent condition. This measurement allowed detection and mapping of dopamine released in each voxel at each time point. By comparing the rate of change measured in the Congruent and Incongruent conditions, we located voxels where it increased significantly during task performance (Incongruent condition). To ensure that this measurement reflected endogenously released dopamine and it was not a chance finding, we measured additional receptor kinetic parameters using the E-SRTM [14]. These parameters included the binding potentials (BPs) and dissociation coefficients (k_2a_) of the ligand in the Congruent and Incongruent conditions. Voxel-wise comparison of the parameter values allowed us to locate voxels where the values (ΔBP and Δk_2a_) changed significantly after task initiation (Incongruent condition).

**Figure 1:**
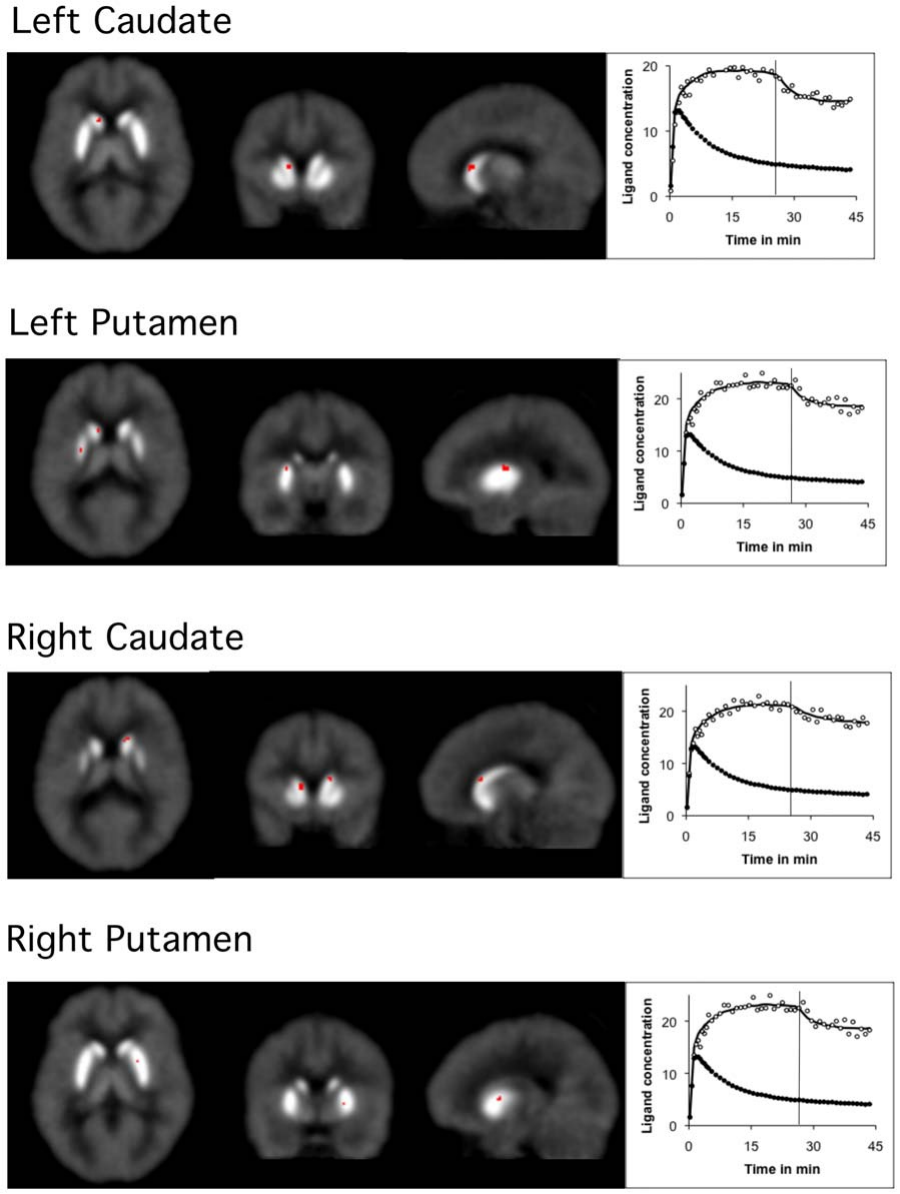
t-maps and time-activity curves showing changes in the rate of ligand dissociation during task performance. The striatal areas where rate of ligand displacement increased significantly in the Incongruent condition of the flanker task are shown on the t-maps. The most significant increase was observed on the dorsal aspect of the body of left caudate. The time-activity curves show the ligand concentration (open circles) and least square fit (solid lines) in a striatal area (upper curve) and in the reference region (lower curves). The data on the left of the vertical lines were acquired in the Congruent condition and those on the right were obtained in the Incongruent condition. Significant reduction in the ligand concentration in the Incongruent condition suggests that the rate of ligand displacement increased during task performance. The increase was due to competitive displacement induced by endogenously released dopamine. There was no significant change in the rate of ligand displacement in the reference region (cerebellum). The time-activity curves were drawn using the mean data acquired from the voxels where maximum changes were observed in each area. This analysis used the linear extension of reference region tissue model (LE-SRRM).

The LE-SRRM analysis revealed that the values of γ changed significantly after task initiation in 4 striatal areas located one each in the caudate and putamen of the two hemispheres (Figure 1). It was most significant (Table 1) on the dorsal aspect of anterior part of the body of left caudate (t = 2.56). Stereotactic (MNI) coordinates (x,y,z) of this location were −10,14, and 8 mm. In this area we observed maximum change in the rate of ligand displacement (γ = 0.1). It was 384% higher than the mean striatal value (0.026). The changes were less significant (t<2.1) in the other three striatal areas: the left dorsal putamen (−22,4,−6); right dorsal body of the caudate (16,16,14); and right dorsal putamen (24,4,2). In these areas values of γ were relatively low (<0.08) but significantly higher than the mean striatal value (Table 1).

**Table 1:** The rate of ligand displacement increased significantly after task initiation (Incongruent condition) in four striatal areas.

Region	MNI (x,y,z)	t-value of ΔΥ	%ΔΥ	%Δk2
L Caudate	−10; 14; 8	2.58	384	142
R Putamen	24,4,2	2.10	230	157
L Putamen	−22 4 −6	2.04	269	143
R Caudate	16,16,14	2.04	308	119

The values were estimated using linear extension of simplified reference region model (LE-SRRM).

MNI = Montreal Neurological Institute stereotactic coordinates; ΔΥ = change in the rate of ligand displacement after task initiation; %ΔΥ and %Δk2 = %change from the mean values measured in the striatum.

**Figure 2:**
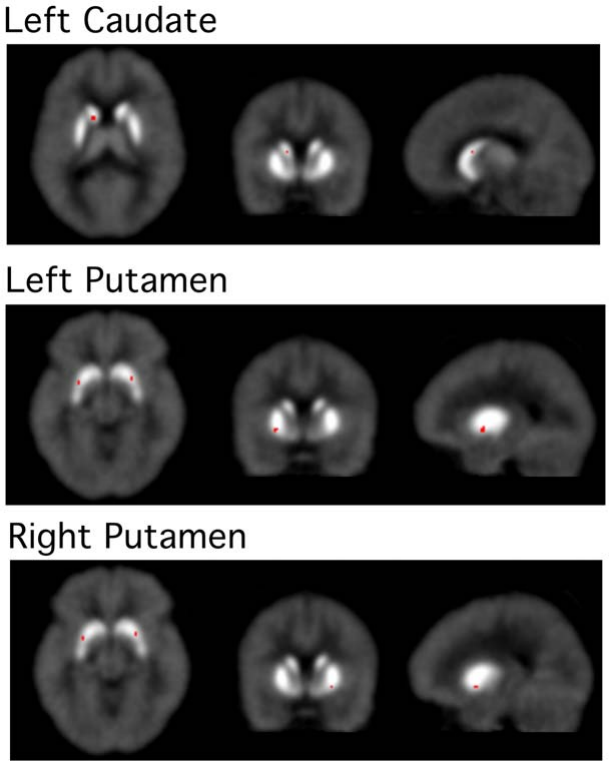
t-maps of ΔBP showing reduction in the ligand binding potential during task performance. The t-maps generated using extended reference region tissue model (E-SRTM) show striatal areas where the ligand binding potential decreased significantly in the Incongruent condition in comparison with the Congruent condition. It was most significant in the left caudate and putamen. These areas are located in close proximity to the locations where increased rate of ligand displacement was observed (Figure 1). An agreement in the data computed using two different receptor kinetic models significantly enhances the reliability of results.

To ensure validity of this finding we estimated the ligand BP and k_2a_ (Table 2) in the Congruent and Incongruent conditions using E-SRTM [14]. As compared to the control (Congruent condition), the BP decreased in 3 striatal areas (Figure 2) during task performance (Incongruent condition). It was most significant (t>2.5) in the left caudate (28%) and left putamen (26%). Additionally, relatively small (23%) but significant (t = 2.21) decrease was observed in the right putamen. There was no significant change in any other area. The ligand dissociation coefficient (k_2a_) also increased in all of these 3 areas but it was statistically significant only in the left caudate (t = 2.07).

**Table 2:** Receptor kinetic parameters measured before (Congruent condition) and after (Incongruent condition) task initiation.

Region	MNI (x,y,z)	BP0 (Cong)	BP1 (Incong)	ΔBP	t-value ΔBP	k_2a_ (Cong)	k_2a_ (Incong)	Δ k_2a_	t-value Δ k_2a_
L Putamen	−26,4, −6	3.85	2.84	26%	2.53	0.048	0.060	20%	1.62
L Caudate	−12;8;10	3.38	2.44	28%	2.51	0.049	0.062	21%	2.07
R Putamen	24,8, −8	3.44	2.64	23%	2.21	0.049	0.059	17%	1.44

The values were estimated using extended simplified reference tissue model (E-SRTM).

MNI = Montreal Neurological Institute stereotactic coordinates; BP0 = ligand binding potential in the Congruent condition; BP1 = ligand binding potential in the Incongruent condition; ΔBP = change in BP after task initiation; t-value ΔBP = t values of the difference in BP before and after task initiation; k_2a_ = dissociation coefficient of the specific binding (k_2_/k_2_-1); Cong = Congruent condition; Incong = Incongruent condition.

Thus, both models found most significant change in the left dorsal caudate. Additionally, both models suggested significant changes in the left and right putamen also. Interestingly, the voxels where maximum change in the rate of ligand displacement (γ) and maximum reduction in the ligand BP occurred, were located within 6 mm of each other in the left caudate and putamen even though these measurements were made using two different receptor kinetic models. In the right putamen these locations were >10 mm apart. It appears that the two models picked up activations from the same neuronal clusters of the left caudate (−10,14,8 and −12,8,10) and left putamen (−22,4,−6 and −26,4,−6). In the right putamen (24,4,2 and 24,8,−8) activations identified by the two models probably came from different clusters. Changes in the left caudate were therefore most significant, consistent and reliable. All parameter values measured in this area were consistent with increased dopamine release in the Incongruent condition in comparison with the Congruent condition.

## Discussion

The results demonstrate increased dopamine release in a number of striatal areas during performance of a flanker task. The increase was most significant on the dorsal aspect of the body of left caudate. All receptor kinetic parameters measured in this area (using two different receptor kinetic models: LE-SRRM and E-SRTM) suggested significant release of endogenous dopamine. In addition, most parameter values suggest dopamine release in three additional striatal locations: dorsal part of the left and right putamen and body of the right caudate (Figure 1).

These findings are interesting because in an earlier fMRI experiment [4] we found increased activation in the same region of the left caudate. Further, the maxima of BOLD response observed in the fMRI experiment and change in the rate of ligand displacement observed in the present experiment were located only 4 mm apart (MNI coordinates: −14,16,10 and −10,14,8). Additionally, maxima of the fMRI activation were located within 8 mm of the location where maximum decrease in the ligand BP (−12,8,10) was observed during task performance. Finding of activation in the same location in experiments that used different techniques validates the observation and underscores significance of the left caudate in processing of executive inhibition.

This observation of dopamine release in the left caudate is consistent with the observation of a number of fMRI studies [15], [16], [17], [18]. These studies however, have implicated other striatal areas also in the processing of executive inhibition tasks, and it was suggested that different striatal structures process different aspects of the task. Thus, caudate and putamen of the right hemisphere are associated with the preparatory phase of response execution [19], [20] and those of the left side with inhibition and interference resolution [18]. In a recent study [18] the caudate and putamen of both hemispheres were activated in a flanker task that involved response selection and interference suppression. When the task was modified to require only response selection without interference (in a stimulus-response compatibility task) only the caudate was activated. Further, requirement of inhibition without selection (in a go-no-go task) activated the right putamen. This finding is supported by another recent experiment in which a negative correlation was observed between the volume of left putamen and the degree of interference. This study also found a positive correlation between the right putamen volume and the accuracy of response [21].

Thus, it appears that different striatal areas process different aspects of the task. The location of striatal activity in an experiment therefore depends on the degree to which these aspects/components are expressed. Thus, in the present experiment interference suppression was the most prominent component and the most significant activation was observed in the left caudate. It therefore suggests that the dopamine system of left caudate is involved in the processing associated with the inhibition of unwanted response.

This suggestion is consistent with the observation of hyperactivity, agitation and inattention (due to loss of inhibitory control) following lesion, destruction or shrinkage of the caudate head [22]. In a recent study impaired executive function in patients with temporal lobe epilepsy has been attributed to the atrophy of left caudate in vicinity of the area where we found dopamine release [23]. It appears that the caudate is able to exert inhibitory control due to its functional connection with the dorsolateral prefrontal cortex (DLPFC) and ACC [24]. In animals dorsal caudate receives cortico-caudate projections from the dorsolateral frontal area and the cingulate [25]. Functional connection between these areas in the human brain has been recently demonstrated in an fMRI experiment [26]. In this experiment simultaneous activation of these areas (the left dorsal caudate, DLPFC and ACC) was observed when attention was focused on a target. Since focused attention is required to resolve interference, the DLPFC and ACC are most consistently activated during resolution of interference caused by multiple response options [27]. In addition to interference suppression, the caudate and its functional connection to the DLPFC are needed to inhibit irrelevant options. It appears that the same frontal areas, located in different hemispheres are activated when the emphasis of task is shifted from response selection to inhibition. These activations are lateralized on the left hemisphere when a task requires response selection and on the right, when the emphasis shifts to inhibition [28]. Further, clinical evidence suggests that the activations associated with inhibition are dependent on dopamine neurotransmission. That is why unmedicated PD patients have difficulty ignoring non-essential stimuli [29], [30], [31].

The neural mechanism that allows dopamine to control inhibition in the human brain is not known but animal studies suggest a possible cellular mechanism. For example, after dopamine neurons are depleted in monkeys, the number of inhibited neurons reduces and the number of nonspecifically activated cells increases significantly [3]. As a result these monkeys find it difficult to select an appropriate response and focus attention on a stimulus. This dopaminergic effect on focused attention is validated in hyper-dopaminergic psychiatric conditions like schizophrenia. These patients are generally hyper-attentive [32], [33] and have difficulty in shifting attention away from irrelevant stimuli. Thus, it appears the inhibitory system works most efficiently when dopaminergic activity is optimal. Both high and low levels disrupt inhibition. This effect is similar to the dopaminergic effect on cognitive functions, which are impaired at both high and low levels of dopaminergic activity [34].

The other striatal areas where changes in dopamine release were relatively small, process aspects of the flanker task that were inadequately expressed in the current experiment. These aspects include selection of an appropriate response. The response selection is an important aspect of not only flanker task but also those of learning and reward systems. The dopamine system is believed to facilitate learning of the outcome of a response and therefore, help us select the most rewarding response [35]. Therefore, dopaminergic agents alter outcome-based selection in PD patients and change their bias for learning from negative outcomes in favor of positive outcome [36]. This observation is consistent with the actor-critic model of reward and reinforcement. The model assumes that the dopamine system learns to select the action that is most rewarding [5].

### Striatal Dopamine and Executive Function

These results provide additional data to help us understand dopaminergic processing of executive function. Previously, dopamine release in the left and right caudate and the right putamen was observed during set-shifting in Montreal card sorting task [37]. We observed dopamine release in the same areas in the current experiment. The similarity is not surprising because set-shifts also involve inhibition – inhibition of the current strategy. In addition to inhibition, set-shift involves selection of a new strategy, which (as discussed earlier) is not strongly expressed in the flanker task. Probably because of this difference, there was a stronger activation of the right caudate during set-shifting. Interestingly, dopamine release in the right caudate is reported also in the spatial working memory [38] and explicit motor memory tasks [10]. Since both of these tasks required volunteers to select a response based on spatial location of a stimulus, it appears that that the dopamine system of right caudate is involved in the selection process. As discussed earlier, dopamine of the left caudate is associated with inhibition. Additionally, the evidence suggests that the dopamine of the right putamen is also involved in inhibition. Therefore, increased dopamine release in this area is observed in the flanker task (current experiment), in the set-shifting experiment and during processing of explicit motor memory task. All of these tasks involve inhibition of unwanted response. These observations are consistent with the BOLD activations observed in the right putamen in a go-no-go task that involved inhibition without response selection [18]. However, we did not find dopamine release in this area in an implicit motor memory task, which also required volunteers to inhibit unwanted response but the inhibition in this experiment was nonconscious [11]. The dopamine system of right putamen therefore is involved in the processing of only voluntary inhibition. It will therefore be interesting to see if dopamine is released in the right putamen during processing of a task involving non-conscious inhibition (e.g. negative priming).

The dopamine system of the left putamen is also involved in the processing of executive function. It is activated in working memory but not in set-shifting task. This system was activated in the current experiment also. In an earlier molecular imaging experiment we found dopamine release in the posterior part of the left putamen during planning and execution of motor responses [10], [11], [12]. Since both, working memory and flanker task (used in the current experiment) involve planning and execution, this activation is consistent with our earlier observations. The anterior left putamen however may have a different function. A significant increase in dopamine release in this area was recently observed following rTMS (repetitive transcranial magnetic stimulation) induced suppression of DLPFC during performance of Montreal card sorting task [39]. This is an intriguing finding because it was observed only when DLPFC activity is suppressed. It indicates that the DLPFC controls dopamine release in the anterior left putamen and that this area takes over some of the functions of DLPFC. It also indicates that the dopamine systems of the structures located inside and outside the striatum interact during processing of the executive function. It is therefore important to study the role of extrastriatal dopamine in executive processing. Unfortunately, in the current experiment we were able to study only striatal dopamine because the ligand ^11^C-raclopride does not bind in detectable amount in the low receptor density areas outside the striatum [13]. Dopamine released in these areas can however be detected using a high affinity dopamine receptor ligands such as ^18^F-fallypride. We recently used this ligand to detect and map dopamine released outside the striatum during emotional processing [9]. Since a number of extrastriatal brain areas are involved in the processing of executive function [4], [18], [27], our understanding of the neurochemical control of human executive function will remain incomplete until dopamine released in extrastriatal areas is characterized.

Thus, the current experiment demonstrates that dopamine is released in a number of striatal areas during processing of a flanker task, which involves inhibition of irrelevant response options. The most significant increase in dopamine release was observed on the dorsal aspect of the body of left caudate. By providing evidence of dopaminergic processing of an important executive function, the results of this experiment will help us define dopaminergic control of the human executive function. Additionally, the study demonstrates that the neurochemical change associated with cognitive processing can be detected and mapped using a single-scan dynamic molecular imaging technique.

## Materials and Methods

### Ethics Statement

This study was approved by Partner's Human Research Committee, Boston, MA 02116. The IRB approved procedure for obtaining written informed consent from each participant was used in the study.

The study was conducted on right-handed healthy young volunteers (n = 10) of either sex (mean age 33.1 years; male 4). None of the volunteers or their first-degree relatives had current or past history of a psychiatric or neurological disorder. Additionally, volunteers had no history of chemical dependency, or use of a dopamine-modifying drug in past 12 months. Pregnant women were not included because of uncertain adverse effect of ionizing radiation on developing fetus. After obtaining IRB approved written informed consent volunteers were positioned on the bed of a positron emission tomography (PET) camera and administered intravenous bolus of a dopamine receptor ligand ^11^C-raclopride (mean dose 13.6 mCi) at a high specific activity (mean specific activity 1159 mCi/micromole). Immediately after the injection volunteers performed a modified version of Eriksen's flanker task [2]. The PET data acquisition also started at the same time. The data were acquired at 30 sec frames during the first 5 min and at 60 sec frames thereafter for the next 40 min, using an ECAT EXACT HR+ PET camera operating in 3D mode.

**Figure 3:**
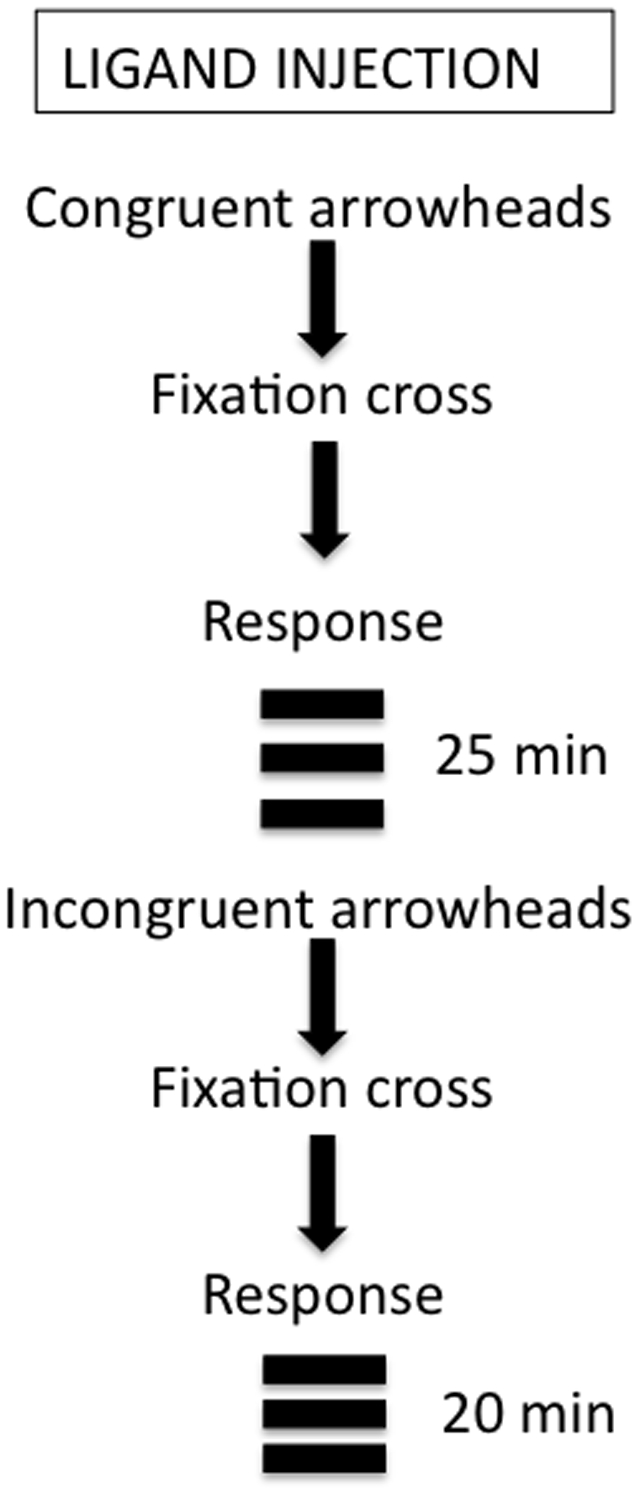
Schematic diagram of the sequence of events.

In the flanker task volunteers were shown a series of 7 arrowheads and asked to press a key using the right index and middle fingers to indicate the direction the arrowhead located in the center (target) was pointing. They were asked to respond as quickly and as accurately as possible. The task had a Congruent and an Incongruent condition. The Congruent condition was started immediately after the ligand injection and in this condition all arrowheads pointed to the same direction (e.g., >>>>>>> or <<<<<<<). After 25 min, unbeknownst to volunteers, this condition was terminated and the Incongruent condition started. In this condition direction of the target arrowhead was changed so that the flanker and target arrowheads pointed to opposite directions (e.g., >>><>>> or <<<><<<). The Incongruent condition was administered for 20 min and in each trial the stimulus was presented for 800 msec. It was followed by a cross mark for 1900 msec (Figure 3). There was a 15 sec break after every 4 min. The response time and accuracy of responses were recorded in each trial and the ligand concentration was dynamically measured during entire scan session.

The PET data were analyzed using methods used in our earlier experiments [8], [9], [10], [11], [12]. The analysis involved measurement of receptor kinetic parameters using modified versions of the simplified reference tissue model or SRTM [40]. Based on these parameter values, the rate of ligand displacement (from receptor sites) was estimated dynamically through out the experiment to locate striatal areas where dopamine was released during task performance. We also estimated the ligand binding potential (BP) in the Congruent and Incongruent condition. These estimates (along with the other receptor kinetic parameters) were used to detect and map dopamine released during task performance.

**Molecular Imaging and PET Data Analysis:** The dynamic molecular imaging technique exploits the competition between an injected radioligand and endogenously released neurotransmitter for occupancy of receptor binding sites [7]. Because of this competition dopamine released during task performance displaces the ligand from receptor sites and reduces its BP. Using receptor kinetic models, the displacement rate, BP and other receptor kinetic parameters are dynamically measured in this technique to detect and map dopamine released during task performance. In previous experiments we used this method to study dopamine release during performance of a number of cognitive, emotional and behavioral tasks [8], [9], [10], [11], [12].

To measure receptor kinetic parameters, the PET data were analyzed using the following steps: First, images were reconstructed as 128×128×63 element volumes using a standard three-dimensional filtered back projection algorithm with corrections for photon attenuation, random coincidences, scatter, and dead time. To minimize residual effects of head movement, images were registered to align each frame to a common orientation. This was accomplished by realigning all frames to a reference frame (the frame acquired at 25 min). Thereafter, a mean image of the first 25 minutes' acquisition was created and used as the source image for spatial normalization, employing a raclopride template (which matched the MNI template) developed in our laboratory. All frames were then smoothed using a 5 mm FWHM Gaussian filter. The routines of statistical parametric mapping software (SPM8; Wellcome Department of Imaging Neuroscience, London) were used for realignment, spatial normalization, and smoothing. Thereafter, voxel-wise analyses were carried out on realigned, normalized and smoothed images to estimate receptor kinetic parameters in each subject. The analysis used receptor kinetic models designed to detect transient change in kinetic parameters. These models are described in earlier publications [12], [13], [14] and explained briefly in the following paragraphs. Parameter values were computed in each voxel at each time point to locate the areas where values changed significantly after task initiation (i.e. in the Incongruent condition). Additionally, time-activity curves were drawn for the voxels showing maximum ligand displacement. These computations used the cerebellum as a reference region and assumed negligible density of dopamine receptors in this region. A time-activity curve for the cerebellum was also drawn to estimate clearance rate of the free and nonspecifically bound ligand. Thereafter, the kinetic parameters (including the ligand BP) were measured in each condition separately in each volunteer. Individual values were then pooled to acquire cohort mean of each parameter value in each condition. By comparing values measured in the Congruent and Incongruent conditions, we located voxels where the values (and ligand BP) changed significantly after task initiation (Incongruent condition). Thus multiple receptor kinetic parameters were used to detect and map dopamine released during task performance.

### Kinetic Models

We used the linear extension of simplified reference region model or LE-SRRM [12], [13], and the extended simplified reference tissue model or E-SRTM [14] to measure receptor kinetic parameters. Both models are modified forms of the SRTM [40], which was developed to measure time dependent changes in receptor kinetic parameters. There was a need to modify the SRTM because it assumes steady physiological state throughout the experiment. This assumption is not consistent with the design of the single-scan method used in this study. Since task condition was changed from Congruent to Incongruent in the current experiment, the steady state was not maintained. The assumption of steady state was eliminated in the LE-SRRM and the E-SRTM using different approaches. The LE-SRRM allows the dissociation rate of ligand to change in response to an altered synaptic level of neurotransmitter by introducing a term 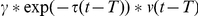 in the dissociation parameter of SRTM. In this term, γ represents the rate of change in ligand displacement, τ allows gradual recovery of kinetic parameters after initial rapid release of dopamine, t denotes the measurement time, T is the time of change in neurotransmitter level, and ν is the unit step function.

The analysis using the LE-SRRM involved measurement of the values of receptor kinetic parameters and γ on a voxel-by-voxel basis using the least squares fitting procedures. The null hypothesis assumed that the task did not elicit dopamine release and there was no change in the rate of ligand displacement after task initiation. This hypothesis was tested in each subject and values of the displacement parameter γ were pooled across subjects to acquire a cohort mean and variance. Additionally, parameters that describe ligand transport and binding, and the time dependent effects elicited by the task were also estimated. The differential equation describing the model for the instantaneous concentration history of the ligand can be expressed as:
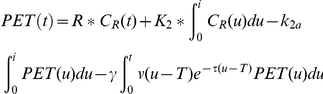
where, C_R_ is the concentration of radioligand in a region devoid of specific binding (reference), PET is the concentration of radioligand in a voxel with specific binding, R is the ratio of transport rates in the tissue and reference regions, k_2_ describes clearance of nonspecifically bound tracer from the voxel, and k_2a_ includes information on dissociation from the receptor, t denotes measurement time, τ allows gradual recovery of kinetic parameters, T is the task initiation time and ν(u-T) is the unit step function.

The E-SRTM [14] uses a different approach to account for a change in physiological state induced by switching task conditions. It assumes that the two conditions (i.e., Congruent and Incongruent) are two separate datasets: one acquired before (Congruent condition) and the other after (Incongruent condition) an intervention. Since steady state was maintained within each condition, the SRTM could be applied to each of these datasets. This assumption allows measurement of receptor kinetic parameters in each condition. By comparing parameter values measured in the two conditions in each voxel, dopamine released during task performance was detected and mapped.

We used differential equations and solutions of the E-SRTM [14] to measure the ligand BP and other kinetic parameters in the Congruent and Incongruent conditions. These values were measured at the voxel level to allow accurate mapping of endogenously released dopamine. For these computations we modified the original E-SRTM and included a bounded non-linear optimizer routine [41] instead of a non-bounded routine, the Marquardt algorithm [42]. This modification allowed us to limit non-physiological solutions. For instance, we bound the solution values of BP between 0 and 6 to prevent the possibility of finding a solution that is outside the physiological range. To ensure reliability of this modification we ran a computer simulation in which tissue and reference region time activity curves were drawn using bounded and non-bounded routines. We found essentially identical values for all 4 kinetic parameters: R_1_, k_2_, BP0 (BP in the Congruent condition) and BP1 (BP in the Incongruent condition). The values of R_1_, k_2_, BP0 and BP1 were 0.95; 0.25; 2.31 and 2.19 respectively using non-bounded routine and 0.95; 0.26; 2.31 and 2.20 respectively with the bounded routine.

The LE-SRRM and E-SRTM differ not only in methods used to eliminate the assumption of steady state, but also in approach for detection of dopamine released during task performance. While LE-SRRM assumes that a change in dissociation coefficient (k_2a_) of the ligand is a sensitive indicator of endogenously released dopamine, the E-SRTM assumes that dopamine release can be detected more accurately by measuring changes in the ligand BP. Furthermore, whereas the LE-SRRM assumes that the receptor kinetic parameters return to the original state in about 10 minutes if task remains unchanged, the E-SRTM makes no such assumption. Since the two models use different approaches to detect dopamine, we used both models to enhance reliability of data analysis. To reconcile findings of the two models, we identified blobs (>5 contiguous voxels) that were ‘activated’ after task initiation in each model analysis. A blob was considered ‘activated’ if a) there was a significant change (p<0.05) in values of γ (estimated using LE-SRRM) after task initiation; b) the ligand BP (measured using E-SRTM) was significantly lower (p<0.05) in the Incongruent condition; c) there was at least 15% increase in dissociation coefficient (k_2_) measured using E-SRTM in the Incongruent condition; and d) maxima of the blobs were located within 6 mm (in all three directions) of each other (to account for Gaussian smoothing in the processing). Thus, we used multiple kinetic parameters and approach to ensure validity of results. Software to implement these models was developed using Matlab (MathWorks, Natick, MA) utilizing the constrained minimization routine of its optimization toolbox.

The LE-SRRM was used as the primary kinetic model because validity of this model has been extensively studied [11], [13]. Further, we used simulations to examine effect of task-induced increase in regional cerebral blood flow (rCBF) on estimated values of the receptor kinetic parameters. These simulations indicated that changes in rCBF do not significantly affect parameter values that were used to estimate dopamine release, unless it is more than 120% of the original rCBF [13]. Since rCBF changes during cognitive task performance are much smaller [43], these changes are not likely to have significant effect on reported results. During performance of a flanker task we observed less than 0.3% change in the MR signal intensity in a previous experiment [4].
